# Abdominal pain as a novel manifestation in children with PCDH19-related epilepsy: A case report

**DOI:** 10.1097/MD.0000000000041211

**Published:** 2025-01-10

**Authors:** Wandong Hu, Fen Zhao, Ying Ren, Hongwei Zhang, Xiaoying Li

**Affiliations:** a Epilepsy Center, Children’s Hospital Affiliated to Shandong University/Jinan Children’s Hospital, Jinan, China; b Department of Neonatal, Children’s Hospital Affiliated to Shandong University/Jinan Children’s Hospital, Jinan, Shandong, China.

**Keywords:** abdominal pain, case report, epilepsy, *PCDH19*, phenotypes

## Abstract

**Rationale:**

PCDH19-related epilepsy manifested various clinical features, including febrile epilepsy, with or without intellectual disability, and psych-behavioral disorders. However, there are few studies demonstrating abdominal pain as the first symptom.

**Patient concerns:**

A 3-year-old Chinese girl presented with clustered seizures of fever sensitivity accompanied by abdominal pain.

**Diagnoses:**

After ultrasonography ruled out abdominal organic lesions, electroencephalographic (EEG) identified abdominal pain was a seizure feature. Trio whole-exome sequence demonstrated a *de novo* and heterozygous *PCDH19* missense mutation (NM_001184880: c.824A>G, P.Y275C), which was confirmed by Sanger sequence. The final diagnosis were “PCDH19-related epilepsy; abdominal pain.”

**Interventions:**

At first, she was treated ineffectively by levetiracetam and valproate. Finally, she was provided with topiramate (TPM).

**Outcomes:**

The patient had gained seizure-free, and the follow-up EEG discharges were reduced.

**Lessons:**

Abdominal pain is a rare autonomic symptom in the setting of seizures. This report describes abdominal pain as a novel manifestation of PCDH19-related epilepsy and might expand its phenotypes spectrum. It also alerts us to perceive the abdominal pain characterized by seizures and early conduct EEG examination to clarify the nature of abdominal pain.

## 1. Introduction

PCDH19-related epilepsy, caused by pathogenic germline variants of Protocadherin 19 (*PCDH19*) gene, is an X-linked, female-predominant, developmental epileptic encephalopathy.^[1]^ Recently, *PCDH19* has been reported as one of the top 10 genes implicated in monogenic epilepsy.^[2]^ It generally affects females and is characterized by infantile-onset seizures that occur in clusters and varying degrees of intellectual disability.^[3,4]^ Patients initially present with difficult-to-control seizures that are often triggered by fever and occur in clusters of multiple seizures in a day. Although multiple seizures are frequently reported with PCDH19-related epilepsy, initial presentation as frequent abdominal pain in children has not been well reported.

Herein, we present a clinically challenging case of seizures characterized by abdominal pain in a female young child with a pathogenic variant in *PCDH19* gene. An interesting phenomenon is that it presented the phenotype of frequent abdominal pain as a seizure type of PCDH19-related epilepsy. The case is extremely important for expanding the latest symptoms of PCDH19-related epilepsy and providing potential reference value for clinical diagnosis.

## 2. Case presentation

This case presentation was approved by the authors’ institutional review board, and consent was obtained from the legal guardian of patient. A 3-year-old girl was admitted to our hospital for a detailed investigation of frequent abdominal pain. She was born of natural birth and had no history of asphyxia or hypoxia. Her intelligence, language, and movement were generally normal, with a Wechsler Intelligence Test score of 83. She was the first and only child of unrelated Chinese parents. There was no family history of seizures or intellectual disability. Her seizure onset at the age of 8 months. She started seizures for 3 to 5 days cluster seizures with upturned eyes or unilateral strabismus, accompanied by postural maintenance of 1 or both limbs lasting between 30 seconds and 2 minutes. Sometimes, she presented cluster febrile seizures onset, then gradually evolved into non-febrile convulsions. The cluster seizures usually recurred with or without a fever in 1 to 2 months. At the age of 11 months, she was diagnosed as epilepsy, then received antiseizure treatments, including sodium valproate and levetiracetam. However, the treatment effects were limited. At the age of 1 year and 5 months, she developed intermittent episodes of abdominal pain, obviously around the umbilicus, and its frequency and degree of episodes gradually increased. And no other epilepsy-related symptoms of abdominal pain were found. The abdominal pain could not be relieved after gastrointestinal related treatment, and inspection of abdominal ultrasound showed no obvious abnormalities. Meanwhile, cranial magnetic resonance imaging (MRI) showed that the left temporal horn was slightly larger, and the rest of the brain was normal. Magnetic resonance spectroscopy (MRS) showed elevated Cho peak and reduced NAA/Cho + Cr ratio in the head and amygdala regions of the hippocampus bilaterally. It was unclear what the essence of abdominal pain in the patient and whether it was a novel symptom of the original illness.

**Figure 1.**
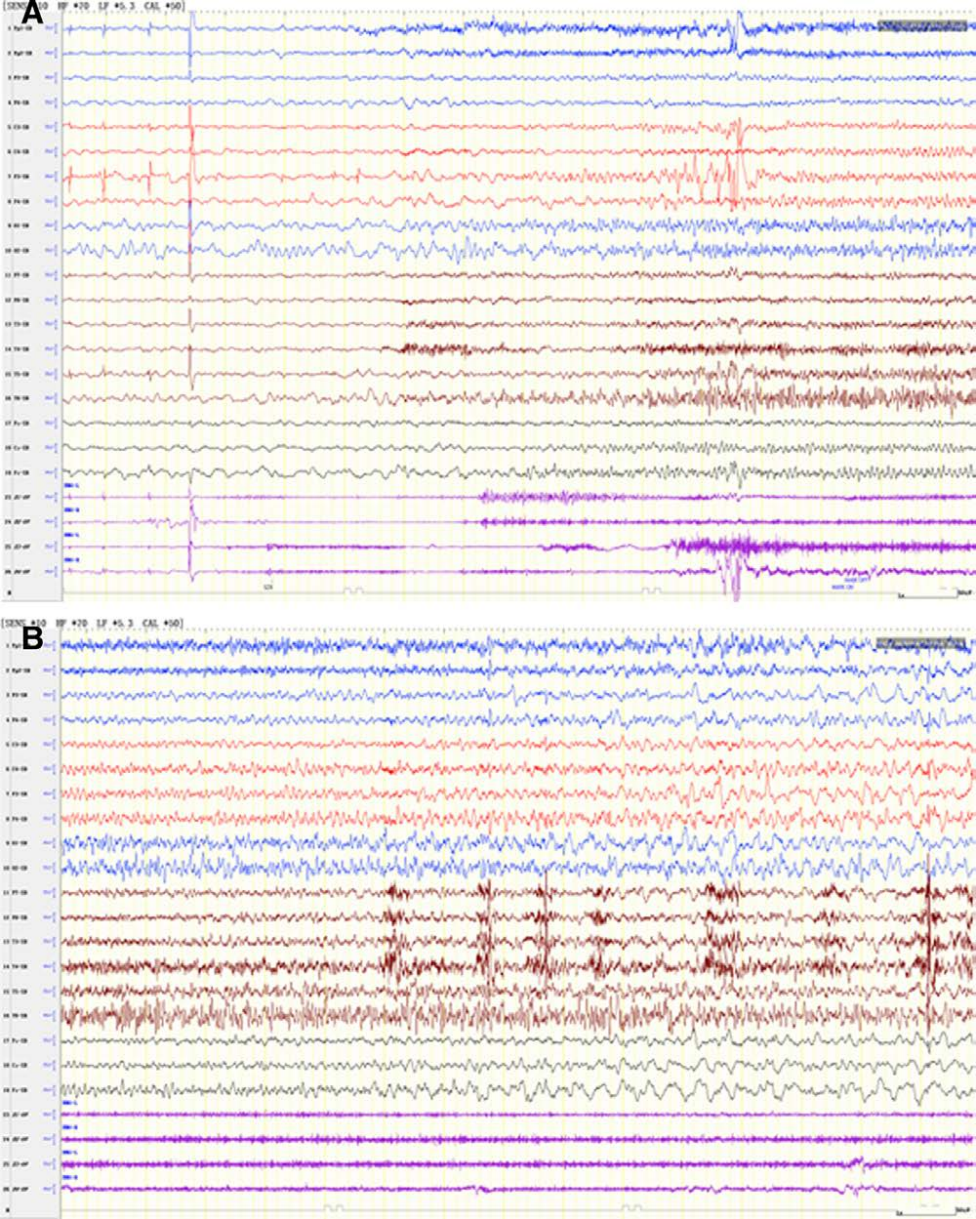
The form of abdominal pain episodes monitored in EEG. EEG = electroencephalographic.

Therefore, we performed a 1-hour video electroencephalographic (VEEG) examination of the patient. The result showed numerous sharp waves and sharp slow wave synchronous/asynchronous discharges in the parietal and mid parietal regions bilaterally, and 5 focal seizures were monitored corresponding to spontaneous abdominal pain, and electroencephalographic (EEG) onset at the apical midline (Pz; Fig. 1). Therefore, the abdominal pain was identified as a seizure type. Dexamethasone administration was initiated for 3 days, and topiramate was added. Her seizures were reduced gradually and the symptoms of abdominal pain disappeared. At the latest follow-up in November 2023, she has been seizure-free for 18 months and EEG monitoring identified no abnormalities.

**Figure 2.**
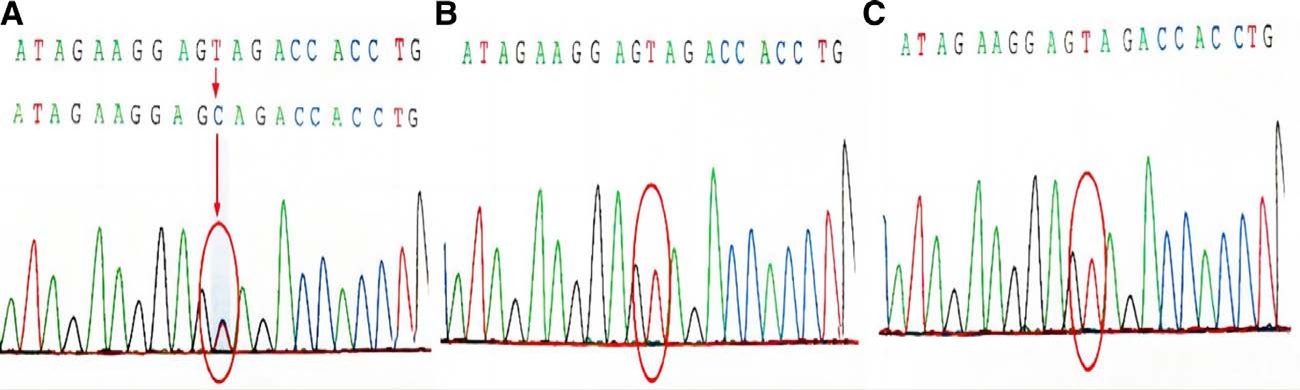
The result of WES in the patient’s family. WES = whole-exome sequence.

Trio-based exome sequencing was performed as described previously and demonstrated a de novo heterozygous variant in PCDH19 (NM_001184880: c.824A > G: p.y275c). This variant was classified as likely pathogenic based on the American College of Medical Genetics variant classification guideline. Mutation screening of her parents showed no mutations were found in her parents in Sanger sequencing (Fig. 2).

## 3. Discussion

We report the case of a young girl with PCDH19-related epilepsy who presented with frequent abdominal pain identified as a novel seizure type of *PCDH19* gene variants. Although fever is reported as a trigger for seizures in girls with PCDH19-related epilepsy, initial presentation of abdominal pain in children has not been previously reported in the context of PCDH19-related epilepsy. Our report expanded the phenology spectrum of PCDH19-related epilepsy and provided potential reference value for the clinical diagnosis.

Protocadherin 19 belongs to the non-clustered delta protocadherin subgroup within the cadherin superfamily, which is primarily expressed in the central nervous system in early brain development.^[1]^ The causative gene PCDH19, which encodes protocadherin 19, is located on chromosome Xq22.1, a region subjected to X-chromosome inactivation and plays an important role in neuronal connections and signal transduction.^[5]^ PCDH19 genomic variants, generally involving the exon1, are correlated with the developmental and epileptic encephalopathy. The PCDH19 mutations mainly manifested a unique X-linked inheritance pattern. According to the previous literature review, epilepsy caused by PCDH19 variants occurs predominantly in heterozygous females and occasionally in mosaic males, whereas hemizygous males are unaffected.^[6]^ The widely recognized pathogenic phenomenon was that the coexistence of mutant and wild-type PCDH19 cells in heterozygous females and mosaic males could interrupt cell–cell communication, and results in cell interference.^[6–8]^ However, hemizygous males have only homologous PCDH19 mutant cells, and could not experience the cell interference. Thus, these males are unaffected.

Mutations in the PCDH19 gene are of incomplete penetrance,^[9]^ which contributes to the phenotypic variability among patients. A number of studies have shown that pathogenic variants in PCDH19 caused a range of neurodevelopmental disorders, including refractory seizures, intellectual disability (ID), autism spectrum disorder (ASD), and behavioral dysregulation.^[4,10]^ Abdominal pain as an autonomic seizure was very rare and often misdiagnosed. In a review, abdominal pain was reported in only 10 (1.7%) of 604 patients with focal epilepsy.^[11]^ In PCDH19-related epilepsy, the phenotype of abdominal pain has not been well reported. Previous studies have indicated that paroxysmal abdominal pain in epilepsy was closely related to insular lobe, amygdala and other structures of the brain.^[11,12]^ Patients with abdominal epilepsy usually have specific EEG abnormalities, such as a series of slow waves, spikes, sharp waves and other abnormal discharge forms.^[13]^ In our case, the interictal discharges and seizure onset in the EEG were all in the apical midline, which speculated that the seizure initiation might be from the insula lobe based on symptomatology combined with EEG analysis.

Interesting, the cluster of acute abdominal pain of our patient was relieved quickly by low-dose corticosteroids. Actually, a previous study has mentioned that the use of corticosteroids in the acute phase of PCDH19-related epilepsy could relieve seizures, but seizures would reoccur within a few weeks.^[14]^ The phenomenon may be attributed to that the neurosteroid allopregnanolone, one of the most potent GABA (γ-aminobutyric acid) receptor modulators, is deficient in female patients with PCDH19 variants because of the downregulation of neurosteroid-metabolizing enzymes.^[15]^ A multicentral cohort study from China showed the increasing allopregnanolone after puberty may be responsible for the reduction in seizure frequency in PCDH19-related epilepsy after the age of 10 years.^[2]^ Therefore, when encountering the seizure type of abdominal pain in clinical practice, low-dose corticosteroids could be considered to relieve acute symptoms when difficult to choose anti-seizure medications (ASMs).

## 4. Conclusion

Abdominal pain is a rare autonomic symptom in the setting of seizures. our report described a girl with abdominal pain as a novel manifestation of PCDH19-related epilepsy and might expand its phenotypes spectrum. It also alerts us to perceive the abdominal pain characterized by seizures and early perform EEG examination to clarify the nature of abdominal pain.

## Acknowledgments

We obtained informed consent from the parents of the child, at the same time, we are very grateful to the child and her parents for their contribution to our study.

## Author contributions

**Conceptualization:** Wandong Hu, Fen Zhao.

**Data curation:** Wandong Hu, Fen Zhao, Ying Ren.

**Resources:** Ying Ren.

**Supervision:** Hongwei Zhang, Xiaoying Li.

**Writing – original draft:** Wandong Hu, Fen Zhao.

**Writing – review & editing:** Wandong Hu, Hongwei Zhang, Xiaoying Li.
